# Primary renal well-differentiated neuroendocrine tumors: report of six cases with an emphasis on the Ki-67 index and mitosis

**DOI:** 10.1186/s13000-019-0791-7

**Published:** 2019-02-07

**Authors:** Bohyun Kim, Han-Seong Kim, Kyung Chul Moon

**Affiliations:** 1 Department of Pathology, Seoul National University College of Medicine, Seoul, Republic of Korea; 2 Department of Pathology, Inje University College of Medicine, Goyang, Republic of Korea

**Keywords:** Renal well-differentiated neuroendocrine tumor, Ki-67, Carcinoid tumor, Mitosis, Grade, Prognosis

## Abstract

**Background:**

Primary renal well-differentiated neuroendocrine tumors (WDNETs) also called carcinoid and atypical carcinoid are extremely rare, and little is known about parameters that may predict prognosis at diagnosis.

**Methods:**

Six cases of primary renal WDNET were collected. After reviewing slides stained with hematoxylin and eosin, proportions of each growth pattern were determined. Synaptophysin, chromogranin, CD56, and Ki-67 immunostaining and Ki-67 morphometric analysis were performed.

**Results:**

Patients included three female and three males, mean age was 53.3 years. The mean tumor size was 4.5 cm, three cases were greater than 5 cm. At the time of initial surgery, lymph node and/or distant metastasis was confirmed in two cases. In a third case, no metastasis was initially found, but lymph node metastasis was identified during follow-up. The remaining three cases did not exhibit metastasis. Histopathologically, the renal WDNETs were primarily composed of ribbon-like and sheet-like growth patterns. Most of the tumors were diffusely positive for neuroendocrine markers. Mitotic count was high (≥2/10HPF) in cases with lymph node or distant metastasis but was low (< 2/10HPF) in non-metastatic cases. Furthermore, the Ki-67 index was also higher (≥3%) in the cases with metastases than in cases without metastasis.

**Conclusion:**

Three out of the six primary renal WDNETs demonstrated aggressive behavior and exhibited increased mitotic counts and Ki-67 indices. These results suggest that mitosis and the Ki-67 index could be used as prognostic indicators for renal WDNET.

## Background

Neuroendocrine tumors (NET) can occur anywhere in the human body, but in more than 90% of cases, they are found in the gastrointestinal tract or the respiratory tract [1]. NETs in the genitourinary system are only found in very small numbers, and primary renal NET is extremely rare [2].

Primary renal NETs consist of well-differentiated neuroendocrine tumors (WDNET) and high grade neuroendocrine carcinomas [3]. High grade neuroendocrine carcinomas can be classified into large cell neuroendocrine carcinoma and small cell neuroendocrine carcinoma, according to their histopathologic features. These disease entities present with extensive necrosis and abundant mitotic figures [4–6].

Primary renal WDNET is a disease entity that histopathologically shows hardly any necrosis and exhibits as low mitotic figures [7]. It has an indolent clinical course but occasionally has lymph node metastasis at surgery, subsequently progressing to metastatic disease [8]. WDNET frequently occurs in relation to congenital renal diseases, such as a horseshoe kidney or renal teratomas [9, 10]. Primary renal WDNETs have very low incidence, with fewer than 100 reported cases [11].

NETs in other organs are classified into three grades that provide significant prognostic information [12]. The World Health Organization (WHO) classifies gastroenteropancreatic NETs having fewer than 2 mitoses/10 high power field (HPF) and a Ki-67 index lower than 3% as low grade, those with 2 to 20 mitoses/10 HPF or a Ki-67 index between 3 and 20% as intermediate, and those with greater than 20 mitoses/10 HPF or a Ki-67 index of greater than 20% as high grade. Lung and thymus NETs with fewer than 2 mitoses/10 HPF in the absence of necrosis are classified as low grade, those with 2 to 10 mitoses/10 HPF or foci of necrosis are classified as intermediate, and those with more than 10 mitoses/10 HPF are classified as high grade [13]. However, in primary renal WDNETs, little is known about the prognostic parameters.

In this study, we report six primary renal WDNET cases with histopathologic characteristics and proliferative activity.

## Methods

### Case selection and histopathological review

We searched the computerized database of the Department of Pathology, Seoul National University Hospital and found six cases that diagnosed with primary renal carcinoid or well-differentiated NET between 2005 and 2016. Four cases underwent radical nephrectomy at Seoul National University Hospital, and two cases (case no. 5 and no. 6) were consultation cases. Primary renal mass was detected by abdominal computed tomography and/or magnetic resonance imaging. We reviewed hematoxylin and eosin (H&E)-stained slides to confirm the diagnosis and to identify various histological parameters, including mitotic activity, the proportion of growth patterns, and tumor local extension. We collected the clinical data and pathologic information from the medical records and pathology reports.

This study was approved by the institutional review board of Seoul National University Hospital.

### Immunohistochemistry and morphometric analysis

Immunohistochemical staining for Ki-67 (MIB-1, Dako, Glostrup, Denmark), CD56 (123C3.D5, Cell Marque, Rocklin, CA), synaptophysin (27G12, Novocastra, Newcastle, UK) and chromogranin (5H7, Leica Biosystems, Newcastle, UK) was performed using the Ventana Benchmark XT automated staining system (Ventana Medical Systems, Tucson, AZ).

Morphometric analysis of Ki-67 immunostaining was performed in primary and metastatic lesions for accurate measurement. All stained slides were scanned at 400x absolute magnification using the Aperio scanning system (Aperio Technologies; Vista, CA). Acquired digital pathology slide images were viewed and analyzed with ImageScope analysis software (version 12; Aperio Technologies, Inc.). The whole slide was examined, and an area was considered acceptable for analysis if it contained 500–1000 tumor cells. The Ki-67 index is the ratio of the number of tumor cells with positive nuclear Ki-67 staining to the total number of tumor cells in a designated area. Three areas with the highest positive nuclear staining cell ratio were designated as hot spot areas. These areas were then analyzed, and a mean value of measurements was calculated [14, 15].

## Results

### Clinicopathologic characteristics

The clinicopathologic features of six primary renal WDNET cases are summarized in Table 1.

**Table 1:** Clinicopathologic characteristics of primary renal well-differentiated neuroendocrine tumors

Case	Age/sex	Location	Initial TNM stage	Tumor size (cm)	Metastasis at initial surgery	New metastasis during follow-up (progression free interval)	Follow-up status (period)
1	54/F	Mid-kidney	Confined to the kidney	3.8	No	No	NED (11 yr. 11 mo)
2	47/F	Mid-kidney	Confined to the kidney	2.4	No	No	NED (7 yr. 2 mo)
3	60/F	Lower pole	Confined to the kidney	1.3	No	No	NED (6 yr. 4 mo)
4	52/M	Mid-kidney	Extension renal sinus fat	5.4	Liver & lymph node	No	AWD (3 yr. 6 mo)
5	59/M	Not stated	Confined to the kidney	5.7	No	Lymph node (60mo)	NED (17 yr. 1 mo)
6	48/M	Lower pole	Confined to the kidney	8.1	Liver & bone	Liver & bone (3mo)	AWD (1 yr. 11 mo)

*AWD* alive with disease, *mo* months, *NED* no evidence of disease, *RN* radical nephrectomy, *yr*. years

None of the six cases exhibited other renal malformations or diseases and were diagnosed incidentally without any tumor-associated signs or symptoms. The mean age of the patients was 53.3 years; three patients were female, while three were male. All six cases underwent radical nephrectomy. Clinical follow-up periods ranged from one to 17 years (average, 95 months). At the time of initial surgery, four cases did not show any additional mass on abdominal computed tomography, magnetic resonance imaging or positron emission tomography, and two cases showed multiple small liver or bone nodules. Thus, all six cases were consistent with primary renal tumor. Case no. 4 exhibited liver and lymph node metastases at the time of initial surgery. Case no. 5 did not present with metastasis at the time of initial surgery, but lymph node metastasis was identified 15 years later. Case no. 6 exhibited both liver and bone metastases at the time of initial surgery. The remaining three cases exhibited no metastasis through the time of this study. Case no. 4 underwent preoperative transarterial chemoembolization (TACE) once and postoperative TACE twice. Case no. 5 underwent lymph node dissection. Case no. 6 underwent postoperative TACE three times and radiation therapy for the treatment of bone metastasis. Two of the six patients exhibiting synchronous metastases are still alive with disease. The remaining four patients are still alive with no evidence of disease.

**Fig. 1:**
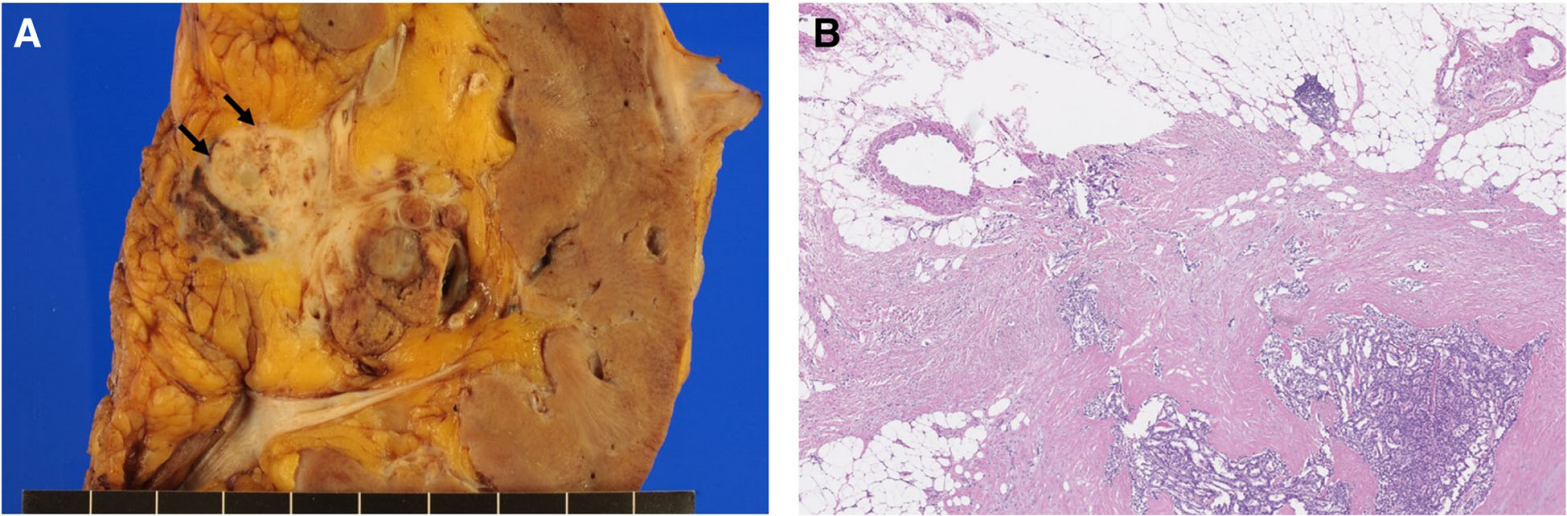
One case exhibited extension to the renal sinus fat on gross finding (**a**) and microscopic finding (**b**, × 40)

All tumors were solitary single masses. Tumor size ranged from 1.3 to 8.1 cm (average, 4.5 cm). Tumor size in all three cases with metastases were greater than 5 cm, and of the three cases without metastasis, tumors were all smaller than 4 cm. The primary tumors in the three cases without metastasis were confined to the renal parenchyma, and extension to the renal sinus fat was observed in one case with lymph node and liver metastases (Fig. 1).

**Fig. 2:**
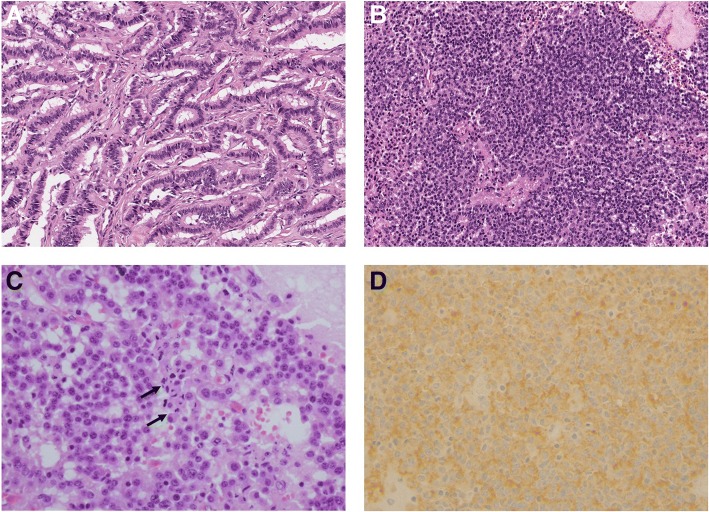
Histologic features of case with lymph node and liver metastasis. Mixed ribbon-like (**a**, × 200) and sheet-like (**b**, × 200) patterns are observed. Also many apoptotic bodies are observed (**c**, × 400). Synaptophysin showed diffuse positive staining (**d**, × 400)

Microscopic examination revealed that histologic features of primary renal WDNET were similar to WDNET in other organs. Monotonous tumor cells have scant to moderate amounts of eosinophilic to clear cytoplasm. Nuclei are round to ovoid with inconspicuous nucleoli and a “salt and pepper” chromatin pattern [16]. Five cases showed a predominantly ribbon-like growth pattern. The remaining case revealed mixed ribbon-like (25%) and sheet-like (75%) growth patterns, and this case had lymph node and liver metastases at the time of initial surgery (Fig. 2a, b). Numerous apoptotic bodies were also observed in case no. 4 (Fig. 2c). None of the six cases exhibited any necrosis. Synaptophysin expression was positive in all six cases, chromogranin expression and CD56 expression were positive in five and three cases, respectively (Table 2 and Fig. 2d).

**Table 2:** Immunohistochemical staining of neuroendocrine markers

Case	Synaptophysin	Chromogranin	CD56
1	P	P	N
2	P	Focal P	P
3	P	Focal P	N
4	P	Focal P	Focal P
5	P	Focal P	N
6	P	N	P

*Focal P* focal positive, *N* negative, *P* positive;

### Proliferative indices

**Fig. 3:**
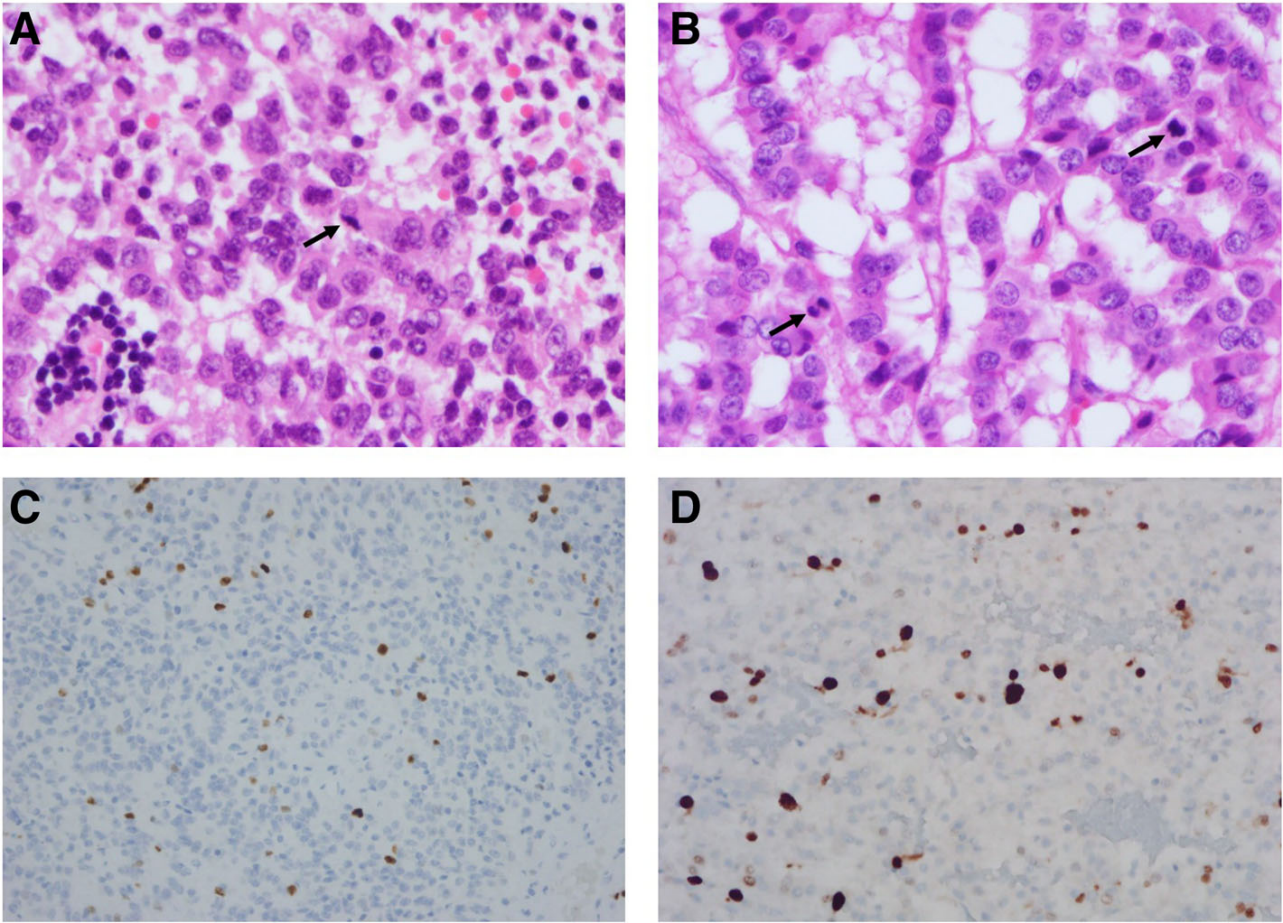
Proliferative indices in cases with metastasis. Mitotic figures (**a**, case no. 4, × 400; **b**, case no. 6, × 400) and Ki-67 immunostaining (**c**, case no. 4, × 400; **d**, case no. 6, × 400) were observed

Mitotic figures were found in cases with lymph node or distant metastasis, while cases without metastasis did not exhibited mitotic figures (Table 3 and Fig. 3a). Metastatic lesions in three cases (case no. 4, 5, and 6) also revealed mitotic figures (Table 3).

**Table 3:** Histologic growth pattern, mitosis and Ki-67 morphometric analysis (Automated counting by analyzer)

Case	Growth pattern: sheet-like/ribbon-like (%)	Mitoses (/10HPF)	Ki-67 index (%)
1 (primary)	3/97	0	0.51
2 (primary)	3/97	0	1.24
3 (primary)	3/97	0	0.6
4 (primary)	75/25	3	8.27
4 (LN)	20/80	2	3.22
5 (primary)	5/95	1	0.66
5 (LN)	5/95	2	3.3
6 (primary)	1/99	6	13.51
6 (Liver)	1/99	4	17.79

*HPF* high power field, *Primary* radical nephrectomy specimen, *Liver* liver biopsy specimen, *LN* lymph node dissection specimen

Results of morphometric analysis are summarized in Tables 3 and 4. Among the three cases with lymph node or distant metastasis, the Ki-67 index of primary lesions was high (≥3%) in two cases and low (< 3%) in one case (Fig. 3b). All three cases without metastasis had a correspondingly low (< 3%) Ki-67 index, while the Ki-67 index of metastatic lesions was also high (≥3%) in all three cases. The average values of mitotic counts and the Ki-67 index were greater in the primary and metastatic lesions of the three cases with metastases than in the primary lesions of the three cases without metastasis (Table 4).

**Table 4:** Comparison of proliferative indices between non-metastatic & metastatic cases

Case	Average mitoses (/10HPF)	Average Ki-67 index (%)
Non-metastatic cases	0	1.12
Metastatic cases (primary lesion)	3.33	7.48
Metastatic cases (metastatic lesion)	2.67	8.1

*HPF* high power field

## Discussion

Renal neuroendocrine tumors are classified into primary renal WDNETs and neuroendocrine carcinomas. Primary renal WDNET includes typical and atypical carcinoid tumors, and neuroendocrine carcinoma includes small cell and large cell neuroendocrine carcinomas [17]. These entities are all International Classification of Diseases for Oncology (ICD-O) behavior 3 code [3]. Primary renal WDNET is known to have distinct histopathological and clinical characteristics, distinct from high-grade neuroendocrine carcinoma [18]. However, little is known about prognostic indicators of tumor aggressiveness, such as lymph node or distant metastasis.

In this study, six cases of surgically resected primary renal WDNET were analyzed. Lymph node or distant metastasis was confirmed in three cases, two of which presented with metastases at the time of initial nephrectomy. Case no. 4 showed lymph node and liver metastases, and case no. 6 showed liver and bone metastases. These two cases with synchronous metastases showed 3 and 6 mitoses/10 HPF on H&E staining, respectively, as well as high Ki-67 indices. Case no. 5 did not show metastasis at the time of initial surgery, but retroperitoneal lymph node metastasis was found 15 years later. In this case, the primary renal lesion showed 1 mitosis/10 HPF on H&E staining and a low Ki-67 index, comparable to the three cases lacking metastasis. All metastatic lesions in case no. 4, 5, and 6 showed high mitotic counts and high Ki-67 indices.

Currently, there is no established grading system for renal NET. However, according to the WHO classification, gastroenteropancreatic, lung, and thymic NETs are classified into low, intermediate and high grades [13].

If the grading system for gastroenteropancreatic NETs was applied to our six cases, two cases (case no. 4 and 6) showing synchronous metastasis would be classified as NET grade 2 due to a high mitotic rate and Ki-67 index. In case no. 5, with non-synchronous lymph node metastasis, the primary renal lesion would be classified as NET grade 1, and the metastatic lymph node lesion would be classified as NET grade 2. The three cases without metastasis would be classified as NET grade 1. If the grading system for lung and thymic NETs was applied, two cases (case no. 4 and 6) showing synchronous metastasis would be classified as atypical carcinoid. In case no. 5, with non-synchronous lymph node metastasis, the primary renal lesion would be classified as typical carcinoid, and the metastatic lymph node lesion would be classified as atypical carcinoid. The three cases without metastasis would be classified as typical carcinoid.

The prognostic value of mitosis and/or the Ki-67 index in gastroenteropancreatic NETs has been well studied [19]. A multi-institutional study reported a positive correlation between the risk of lymph node metastasis and grade in neuroendocrine tumors of the large intestine [20].

Currently, there are no well-defined grading systems in use for renal NET and only a few studies regarding prognostic indicators of primary renal WDNET have been published. Aung et al. reported eleven cases of primary renal neuroendocrine tumors, of which only two cases with liver metastases showed a Ki-67 index of 3 and 5%. They could be classified as NET grade 2, atypical carcinoid, according to WHO classification of lung and thymus tumors. The authors suggested that high proliferative rate could indicate poor outcome [16]. Hansel et al. reported 21 cases of primary renal carcinoid tumor. The case with the highest mitotic figures (4 mitoses/10 HPF) had liver and bone metastases at the time of initial surgery and died of disease eight months later [21]. Raslan et al. suggested that tumor stage, cellular atypia and mitotic figures are likely to correlate with patient outcome [22]. Romero et al. suggested that mitotic figure higher than 1/10 HPF may be related to poor outcome, and tumors of less than 4 cm in size, which are confined to the renal parenchyma, may be related to lower rates of metastasis [1].

In this study, we also revealed that renal WDNETs with metastasis exhibited the higher mitotic counts and Ki-67 indices than those without metastasis. Furthermore, tumor size of the three cases with metastases were greater than 5 cm, while tumor size of the three cases without metastasis were less than 4 cm. A case with extension to the renal sinus fat also presented with lymph node and liver metastases. These results suggested that mitotic count, Ki-67 index and tumor size can be useful prognostic indicators in primary renal WDNET.

## Conclusion

In summary, a higher proliferative activity of primary renal WDNET, including mitotic counts and the Ki-67 index, appears to correlate with the risk of metastasis. The results of our study, in accordance with previous studies, suggest that a grading system based on mitotic counts and the Ki-67 index may be a good candidate for predicting the aggressiveness of primary renal WDNET. Further analysis of a greater number of cases of primary renal WDNET is needed to determine the most appropriate prognostic parameters.

## Abbreviations

H&E: Hematoxylin and eosin
HPF: High power field
ICD-O: International Classification of Diseases for Oncology
NET: Neuroendocrine tumor
TACE: Transarterial chemoembolization
WDNET: Well-differentiated neuroendocrine tumor
WHO: World Health Organization
